# Retrospective analysis of dental implants immediately placed in extraction sockets with periapical pathology: immediate implant placement in infected areas

**DOI:** 10.1186/s12903-023-02986-0

**Published:** 2023-05-18

**Authors:** Sefa Çolak, Mustafa Sami Demïrsoy

**Affiliations:** 1 Tokat Gaziosmanpasa University, Faculty of Dentistry, Department of Oral and Maxillofacial Surgery, Kaleardi Mahallesi Muhittin Fisunoglu Caddesi, Omcalik Sokak, 60030 Merkez / Tokat, Turkey; 2 Sakarya University, Faculty of Dentistry, Department of Oral and Maxillofacial Surgery, Mithatpasa mah, Adnan Menderes Cd. No:122/B, 54100 Adapazarı / Sakarya, Turkey

**Keywords:** Bone guided regeneration, Dental implant, Immediate placement, Periapical lesion, Sinus lifting

## Abstract

**Background:**

The aim of this study is to examine the survival rates of immediate implants placed in extraction sockets with chronic periapical pathology.

**Methods:**

69 patients and 124 immediate implants were included in the study. The patients included in the study were examined in 3 groups. Group 1: Patients who underwent tooth extraction with periapical pathology and immediate implant placement. Group 2: patients who underwent tooth extraction with periapical pathology, immediate implant placement and guided bone regeneration. Group 3: Patients who underwent tooth extraction with periapical pathology, sinus lift procedure and immediate implant placement. In statistical analysis, t-test and Anova analysis were used in the evaluation of quantitative data, cross-tables and chi-square (χ2) test were used in the evaluation of classified qualitative data. Statistical significance was determined as p < 0.05.

**Results:**

It was observed that 116 (95.55%) of 124 implants were successful and 8 (4.45%) failed. The success rate was 97.2% in Group 1, 93.5% in Group 2 and 81.8% in Group 3. A significant correlation was found between the study groups and implant success in terms of χ2 test (p = 0.037). A significant relationship was found between smoking and success in terms of the χ2 test (p = 0.015).

**Conclusions:**

High survival rates are observed for immediate implant placement in sockets with periapical pathology. The success rates observed in guided bone regenerations simultaneously with immediate implant placement are at satisfactory levels. In cases where simultaneous sinus lifting procedures are required, the success rates were observed to be significantly lower. In case of adequate curettage and debridement in sockets with periapical pathology, high implant survival rates are observed. As the complexity of the surgical procedure increases, treatment protocols may progress in safer ways.

## Introduction

Dental implants are an important treatment for restoring function in total or partially edentulous patients. For many years, the standard procedure for dental implant treatment has been to place the dental implant in the healed bone and make it functional after a certain healing period [1]. With the changes observed in implant surgery over time, immediate placement of dental implants in extraction sockets has proven to be a viable and safe treatment option [2–4].

Immediate implant placement has some distinct advantages, such as reducing the number of surgical procedures, shortening the healing process, reducing stress on the patient, and better preservation of soft tissue morphology and alveolar morphology [5–7]. In addition to the advantages, some disadvantages such as the need for the application of regenerative methods can be observed. In addition, the need for the use of bone grafts and barrier membranes may increase due to the incompatibility between the extraction socket and dental implant morphology.

Immediate placement of dental implants in extraction sockets with periapical lesions makes it possible to perform tooth extraction, implant placement and bone regeneration in a single-stage surgery. The disadvantage of the technique is that infection residues in the socket may cause implant contamination during the early healing period [8]. Unlike the conventional method in the application of dental implants, immediate implant applications do not allow the body tissues to complete the infection management. Pathogenic bacteria can be observed in the extraction sites even after extensive irrigation during implant surgery [9]. Comprehensive curettage of granulation tissues and all soft tissue remnants in sockets is required to reduce the inflammatory response [5, 10–12]. In the literature, different techniques such as the use of prophylactic antibiotics to reduce the risk of infection, irrigation with antiseptic agents for decontamination in mechanically hard-to-reach areas, the use of lasers for debridement of extraction sockets before immediate implant placement in extraction sockets are observed [13–16]. Although systemic antibiotic applications in dental implant procedures are controversial, it is observed that different types and doses of antibiotics are prescribed in similar studies [5, 11, 12, 17]. A systematic review evaluating the effect of systemic antibiotic administration on complications in the placement of dental implants generally reports that antibiotics are beneficial in reducing failure in dental implant surgery [18]. Irrigation of extraction sockets with chlorhexidine solutions as an additional procedure for immediate implant placement in infected areas can effectively reduce contamination levels [19, 20].

Although studies showing high success rates for immediate implant placement in sockets with chronic periapical pathology have been published in recent years, the risks of the application still continue to be a matter of debate in clinical practice [17, 21, 22]. The aim of this study is to investigate the survival rates of dental implants immediately placed in extraction sockets with periapical pathology in a specific patient population.

## Materials and methods

The ethical suitability of this retrospective study was approved by the Tokat Gaziosmanpaşa University Clinical Research Ethics Committee (Registration Number: 22- KAEK-046). The study was conducted in accordance with the Helsinki Declaration of Ethics for Medical Research Involving Human Subjects. Verbal and written consent was obtained from all participants or their relatives included in the study, and all participants or their relatives were informed in detail about the study.

### Patients

In the study, the patient population was determined as patients who were operated by a single Oral and Maxillofacial Surgeon (SÇ) between January 2020 and December 2021. The minimum follow-up period after the completion of the prosthetic restoration was 18 months. Existing patient records were reviewed for all patients who had tooth extraction and immediate implant placement. Two researchers (SÇ and MSD) reviewed the radiographs and anamnesis-treatment files of patients who were operated between January 2020 and December 2021. Examination of the presence of periapical pathologies in preoperative radiographs or cone-beam computed tomography (CBCT), stability of the implant, number and location of extracted teeth, number of implants with guided bone regeneration, use of grafts, presence of maxillary sinus lift procedures performed simultaneously with immediate implant placement, presence of implants used Systemic conditions such as height-diameter information, determination of failed implants, smoking, age, gender, implant brand, diabetes and hypertension were determined through patient files and radiography data.

Chronic periapical pathology was defined as periapical radiolucency lesions larger than 3 mm observed in the apical part of decayed teeth, teeth with failed endodontic treatment, and teeth with periodontal disease.

**Fig. 1:**
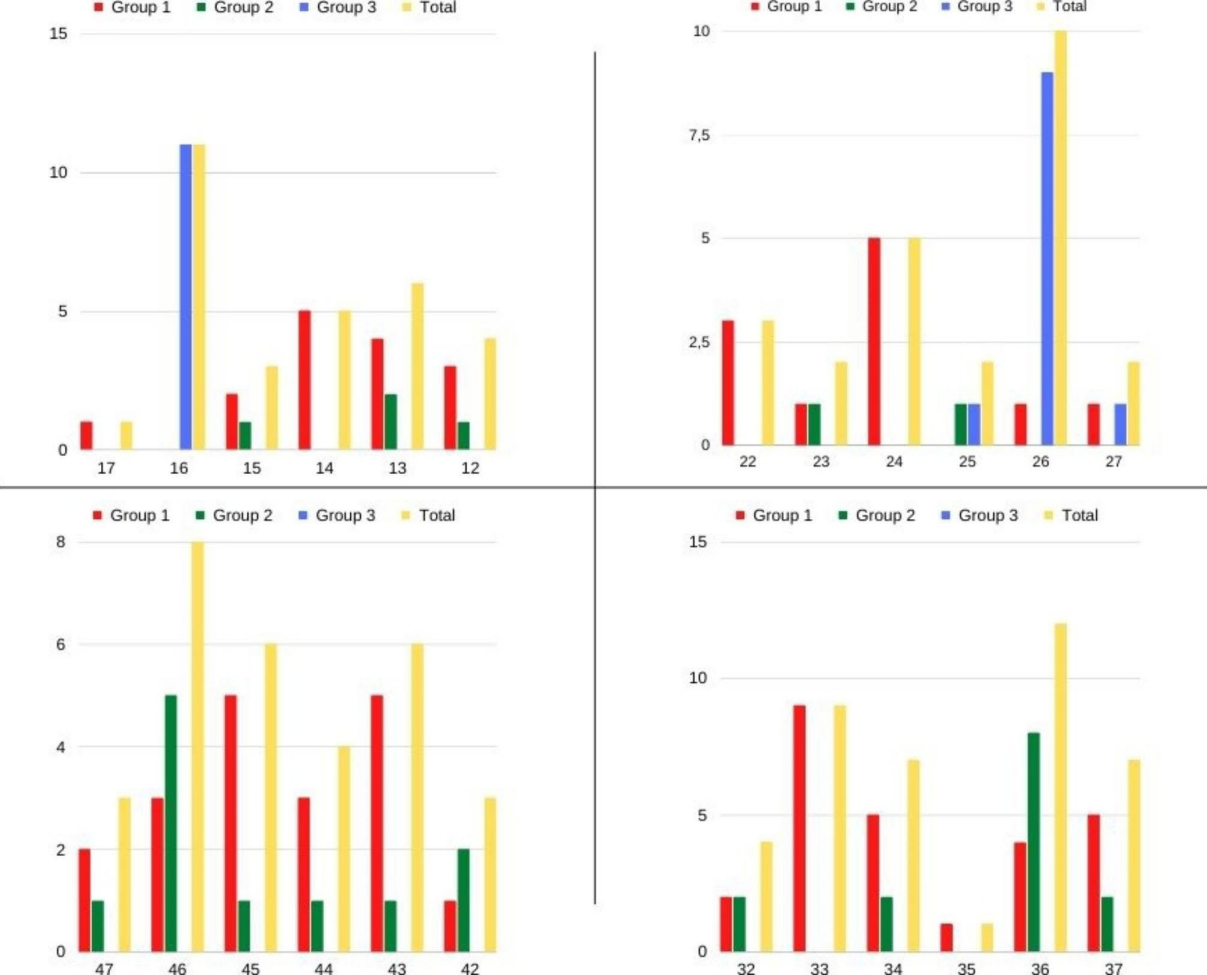
Distribution of implants by region

Exclusion criteria from the study were severely poor oral hygiene, history of previous implant surgery, uncontrolled systemic diseases, severe diabetes mellitus (HbA1C value > 7.5), patients with uncontrolled hypertension, severe osteoporosis (bone mineral density T score < − 2.5), patients using bisphosphonates and derivatives, patients using antiresorptive drugs, patients who received radiotherapy from the head and neck region, psychiatric contraindications, patients whose prosthetic restoration could not be completed, and patients for whom routine clinical-radiographic follow-up and controls could not be performed. Considering the inclusion and exclusion criteria, 69 patients (30 females, 39 males) aged between 24 and 70 years and 124 implants were included in the study (Fig. 1).

### Placement of implants and guided bone regeneration

An application standardization was established for the extraction of teeth with periapical pathology and immediate implant placement in extraction sockets in the patients included in the study. In cases where acute infection was observed simultaneously with chronic periapical pathologies, systemic antibiotic treatment was started 5 days before the preoperative period. Oral Amoxicillin + Clavulanic acid combinations (Amoclavin®-BID, Deva Holding A.Ş, Istanbul, Turkey) were prescribed for routine use. Oral clindamycin applications (Klindan®, Bilim Pharmaceuticals, Istanbul, Turkey) were used in patients with a history of penicillin allergy, and intravenous ampicillin + sulbactam combinations (Devasid®, Deva Holding A.Ş, Istanbul, Turkey) were used in patients who could not take oral medication. Fluctuant abscess foci were drained and 0.12% chlorhexidine (CHX) mouthwash (Chloroben Gargara, Drogsan İlaç, Ankara, Turkey) was prescribed until the day of surgery. Preoperative systemic antibiotics were not prescribed in cases where acute infection was not observed. All patients were given prophylactic antibiotics one hour before the procedure [18]. Intraoral rinsing was performed with 0.12% CHX mouthwash for 1 min before local anesthesia on the day of surgery [19]. After the injection of vasoconstrictor local anesthesia (Ultracain DS forte, AventisPharma GmbH, Vienna, Austria), sulcular incisions and, if necessary, vertical relaxing incisions were made to avoid trauma to the keratinized gingiva during tooth extraction, the flap was lifted and atraumatic tooth extraction was performed. Periapical granulation tissues were removed with a sharp-tipped curette. The extraction socket was first washed with 0.12% CHX solution and then with isotonic saline. After the preparation of the implant socket and the placement of the implant at the subcrestal level (2 mm), the implant was closed with gingival healing abutment at values ​​higher than the determined torque value (40 Ncm). In cases where the torque value was less than 40 Ncm, the cover screw was placed. In patients who underwent guided bone regeneration (GBR) or maxillary sinus lifting (MSL), all implants were closed with cover screws, regardless of torque value. In the postoperative period, systemic antibiotics, Non-steroidal anti-inflammatory drug (NSAID) (Apranax® Fort, Abdi İbrahim, Istanbul, Turkey) for 5 days, and CHX mouthwash three times a day were prescribed to all patients during the 10-day period until the removal of the sutures.

The general idea for immediate implant placement is to graft spaces where the distance between the socket walls and the dental implant is more than 2 mm [23, 24].

After curetting of the periapical granulation tissues and providing the socket preparation, in cases where no defects are observed in the buccal - lingual / palatal walls of the extraction socket and a distance of less than 2 mm is observed between the outer surface of the implant and the socket wall, the remaining spaces after the placement of the implant are not grafted **(Group 1)**.

After implant placement, all spaces were grafted in cases where the distance between the implant and socket walls was greater than 2 mm or there was a defect in the buccal-lingual/palatal walls of the socket **(Group 2)**.

**Fig. 2:**
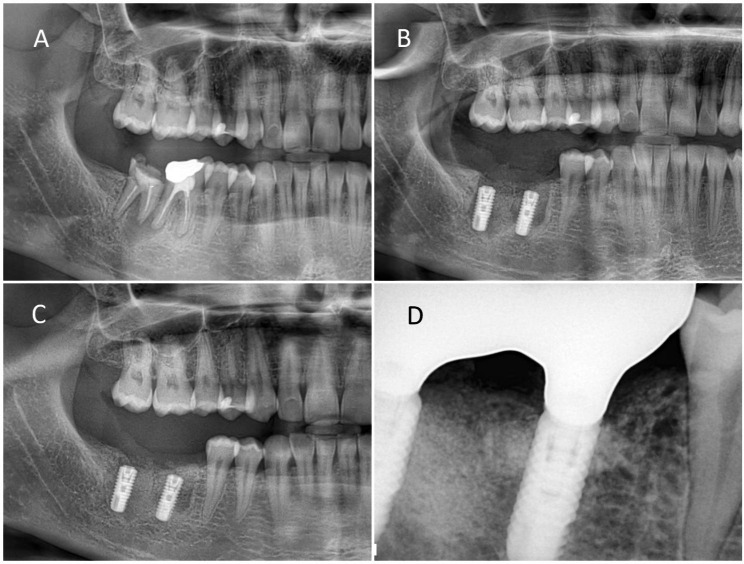
(**A**) Right mandibular first and second molars with chronic periapical pathology due to unsuccessful endodontic treatments. (**B**) Extraction of teeth, placement of immediate implants and filling of socket cavities with guided bone regeneration (1st postoperative week). (**C**) Postoperative 5th month panoramic radiograph image. (**D**) Follow-up radiograph at 12 months after completion of prosthetic restorations

**Fig. 3:**
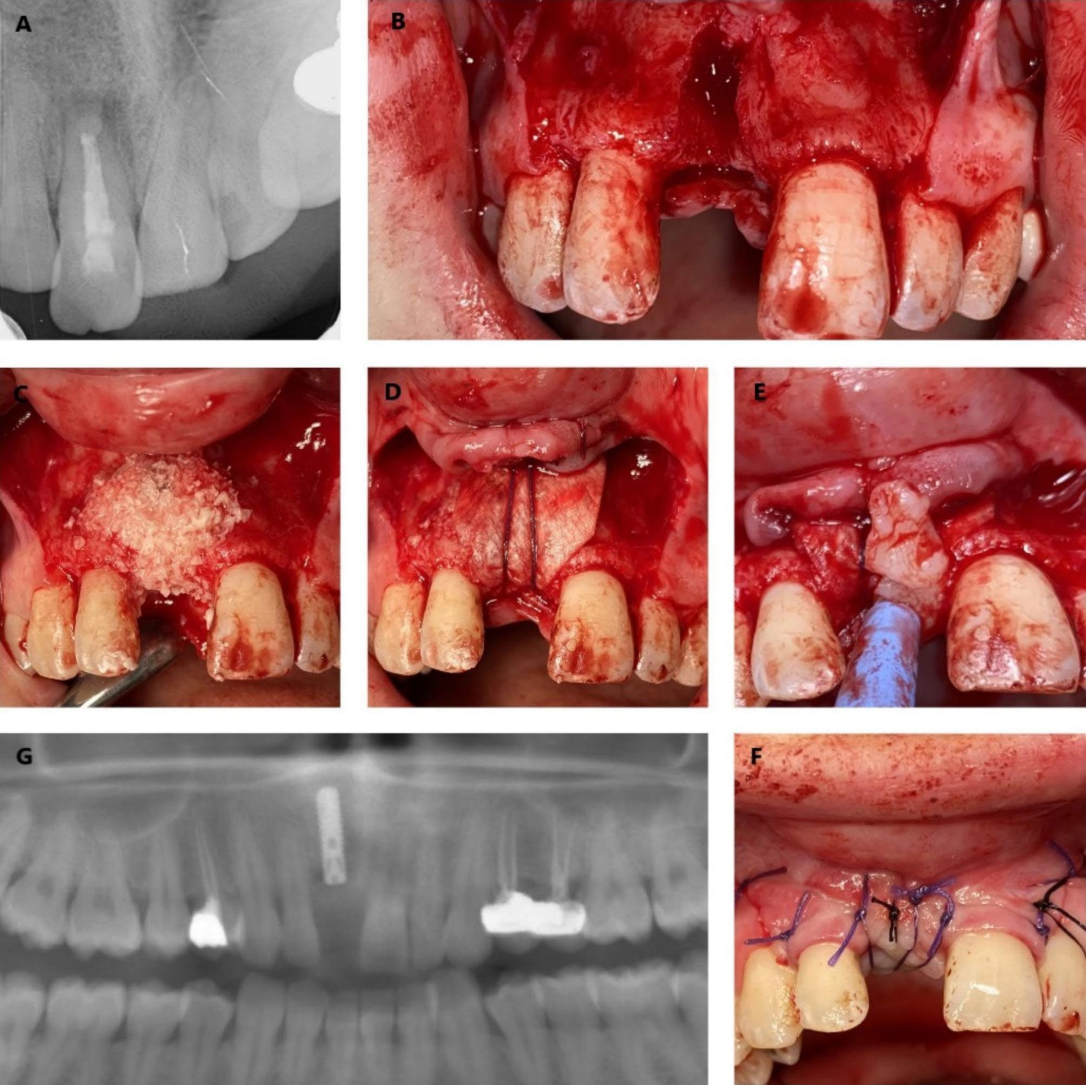
(**A**) Radiographic view of the right maxillary central tooth with periapical pathology. (**B**) Extraction socket after tooth extraction and curettage of periapical lesions. (**C**) Immediate implant placement and grafting of the area. (**D**) Covering the area with a membrane and securing it with a resorbable suture. (**E**) Flipping the flap from the palatal mucosa. (**F**) Primary closure of the area. (**G**) Postoperative radiography

The void spaces in the sockets were grafted with a mineralized cancellous bone allograft (Maxgraft®, Botiss Biomaterials GmbH, Zossen, Germany) (Fig. 2). The augmented areas were covered with a natural collagen membrane (Collprotect® membrane, Botiss Biomaterials GmbH, Zossen, Germany) and the membrane was fixed with a resorbable suture before closing the flaps (Fig. 3). No additional procedure was applied in the areas where the flap could be closed primarily. In the maxilla, especially in the posterior region, a pedicle flap was turned from the palatal mucosa in areas where the flap could not be closed primarily after immediate implant placement. In the mandible, the flap was stretched and the augmented areas were closed primarily using a monofilament suture (Propilen®, Doğsan, Trabzon, Turkey).

### Maxillary sinus augmentation

In the maxillary premolar and molar regions where tooth extractions with periapical pathology were performed, after tooth extraction and curettage of granulation tissues, the lateral approach MSL procedure was performed when the residual bone height was less than 5 mm.

In cases where the residual crestal bone height was more than 5 mm and less than 8 mm, the crestal approach sinus lifting procedure was performed with the use of osteotomes [25]. No additional bone graft was used in patients who underwent the crestal sinus lifting procedure, and these patients were not included in Group 3.

Sinus lifting procedures were not performed in cases where the residual crestal bone height was 8 mm or more after tooth extraction with periapical pathology, and these patients were classified in Group 1.

Sinus lifting procedures in all patients included in Group 3 were performed with a lateral approach with the Dentium Advanced Sinus Kit (DASK)(Dentium, Seoul, Korea). Lateral windows were prepared with an 8 mm diameter diamond round bur. Although the use of piezoelectric devices at this stage offers significant advantages in factors such as minimizing complications and increasing the success rate [25], the use of burs was preferred considering ease of use and accessibility. After the Schneiderian membrane elevation was performed, implant sockets were prepared from the extraction sockets. Before the dental implants were placed, the prepared sinus cavity was rinsed with isotonic saline and covered with a collagen membrane (Collprotect® membrane, Botiss Biomaterials GmbH, Zossen, Germany). The implants were placed and the spaces between the dental implant and the Schneiderian membrane were filled with an allograft (Maxgraft®, Botiss Biomaterials GmbH, Zossen, Germany). In addition, when necessary, the criteria in Group 2 were taken into account in the grafting of the part of the implant in the socket. The lateral window was covered with a collagen membrane and primary closure of the flap was achieved. In cases where primary closure of the flap could not be achieved, the pedicle flap was turned from the palatal region and all flaps were closed with a monofilament suture. Systemic antibiotics, NSAIDs, oral (A-Ferin® Sinus, Bilim İlaç, Istanbul, Turkey) and nasal (Iliadin® Merck, Merck KGaA, Darmstadt, Germany) decongestant and mouthwash containing CHX were prescribed for 5 days postoperatively. Sutures were removed after 15 days and gingival healing abutments were placed 5 months later. In cases where schneiderian membrane perforation was observed during tooth extraction, curettage of periapical pathologies or schneiderian membrane elevation and repair could not be performed, maxillary sinus augmentation and implant placement were postponed to 5 months.

All operated patients were clinically controlled on the postoperative 5th day, 15th day, 1st month, 3rd month and, if necessary, at the 5th month. Radiographic controls were performed on the 15th day, at the 3rd month and, if necessary, at the 5th month. Radiographic evaluations were performed on panoramic and periapical radiographs. In patients who underwent GBR and lateral approach MSL, gingival healing abutments were placed at 5 months, in patients without an additional procedure, gingival healing abutments were placed at 3 months for mandibular implants and at 4 months for maxillary implants. After the completion of the prosthetic restorations, the follow-up period was determined as 1, 3, 6, 12 and 18 months. Certain criteria were considered in accepting osteointegration as successful or unsuccessful [26, 27];

- Implant mobility.
- Suppurative, recurrent periimplant infections.
- Subjective complaints such as pain, foreign body sensation, paresthesia or dysesthesia.
- Large radiolucency areas observed around the implant.
- Bone loss of more than 0.5 mm in the first 6 months after the completion of the prosthetic restoration.

### Statistical analysis

The normality distribution of the study data was checked with the Kolmogorov-Smirnov test before being subjected to statistical analysis. In addition, validation was performed with Skewness and Kurtosis values (from − 1.5 to + 1.5). It was checked whether the variances were homogeneous. While t-test and Anova analyzes were used in the evaluation of quantitative data, cross tables and chi-square (χ2) test were used in the evaluation of classified qualitative data. IBM SPSS 24 (SPSS inc., an IBM Co., Somers, NY) programs were used for analysis. Statistical significance was determined as p < 0.05.

## Results

In the study, 124 implant applications and survival rates in 69 patients, aged between 24 and 70 years (46.92 ± 10.54), who underwent simultaneous immediate implant application with the extraction of teeth with periapical pathology by a single Oral and Maxillofacial Surgeon (SÇ) between January 2020 and December 2021 were evaluated. Of the 69 patients included in the study, 30 (43.48%) were female and 39 (56.52%) were male. The patients included in the study were examined in 3 groups in total.

**Fig. 4:**
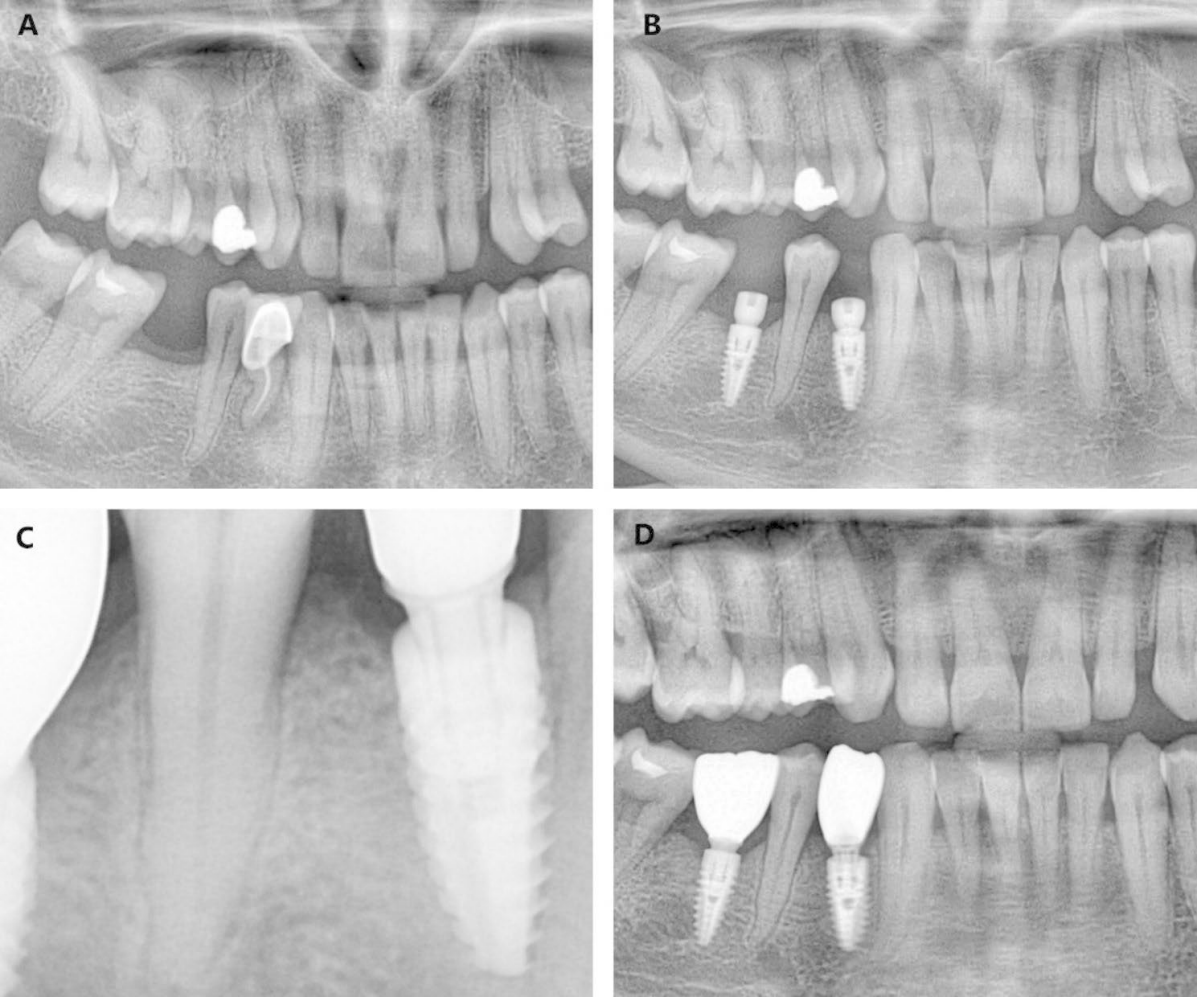
(**A**) Right mandibular first premolar with periapical pathology. (**B**) Placement of the implant after tooth extraction and curettage of the socket. (**C**) Periapical radiography after completion of prosthetic restoration. (**D**) 18. Monthly control radiograph

Group 1, patients whose teeth with periapical pathology were extracted and immediately implanted (71 implants) (Fig. 4).

Group 2, patients who had teeth with periapical pathology extracted, immediate implant placed and GBR performed (31 implants).

Group 3, patients who had teeth with periapical pathology extracted, lateral approach MSL applied and immediate implant placed (22 implants).

The characteristics of all groups and the application procedures in each group are explained in the material and method section. The groups formed were evaluated in terms of the implant brand used, implant length and diameter, patient age, gender, and smoking.

### Implant characteristics (diameter / height), age and smoking, MSL & GBR groups

The number of immediate implants (Group 1) placed after tooth extraction with periapical pathology was 71 (57.26%), the number of implants (Group 2) with simultaneous GBR with the immediate implant was 31 (25%), the number of implants (Group 3) with simultaneous lateral approach MSL with the immediate implant was 22 (% 17.74). The mean diameters of the implants for groups 1,2 and 3 were 4.05 ± 0.495, 4.33 ± 0.513, 4.40 ± 0.334, respectively. The mean lengths of the implants for groups 1,2 and 3 were 11.04 ± 1.855, 10.87 ± 1.668, 8.73 ± 1.202, respectively. A statistically significant relationship was observed between the groups in terms of both implant diameter and implant length (p = 0.002, p = 0.000) (Table 1). The comparisons of the groups within themselves were examined with the Post Hoc test. It is observed that there are differences in the diameters of the implants used between Group 1 and Group 2 and between Group 1 and Group 3. The diameters of the implants used in Group 2 and Group 3 patients are larger than the diameters of the implants used in Group 1 patients. It is observed that there are differences in the lengths of the implants used between Group 1 and Group 3 and between Group 2 and Group 3. Longer implants were used in Group 1 and Group 2 patients than in Group 3 patients. There was no statistically significant relationship between the groups in terms of smoking (p = 0.684) (Table 1). When the mean ages between the groups are examined, it is observed that the mean age is 48.97 ± 8.762 in Group 1, 44.10 ± 12.926 in Group 2, 44.27 ± 11.106 in Group 3. A statistically significant relationship was observed between the groups and age (p = 0.042) (Table 1).

**Table 1:** Distribution of Groups, Implant Diameter and Length Characteristics, Smoking

Variables		Number of Implants	Mean	Std. Deviation	P Value	Post Hoc
Diameter	Group 1	71	4,05	0,495	0,002*	Group-1/2 (0,039) +
	Group 2	31	4,33	0,513		
	Group 3	22	4,40	0,334		Group-1/3 (0,009) +
	Total	124	4,18	0,497		
Length	Group 1	71	11,04	1,855	0,000*	Group-1/3 (0,000) +
	Group 2	31	10,87	1,668		
	Group 3	22	8,73	1,202		Group-2/3 (0,000) +
	Total	124	10,59	1,909		
Smoking (Number/Day)	Group 1	71	8,38	10,649	0,684	
	Group 2	31	6,94	10,302		
	Group 3	22	6,59	7,775		
	Total	124	7,70	10,069		
Age	Group 1	71	48,97	8,762	0,042*	Group-1/2 (0,092) +
	Group 2	31	44,10	12,926		
	Group 3	22	44,27	11,106		
	Total	124	46,92	10,545		

* The Anova test defines the p value. p < 0.05

+ Post Hoc test p value defines the meaning between groups.

### Implant brands and distribution by groups

In patients included in the study, NucleOSS™ T6 (Nucleoss, Şanlılar Ltd ŞTI, İzmir, Turkey) (21, 16.94%), Implance (Implance, AGS Medikal, Trabzon, Turkey) (28, 22.58%), NEODENT TEC-2 ( Neodent, Curitiba, Brazil) (29, 23.39%), Medentika® Microcone (Medentika, Germany) (19, 15.32%) and NTA® Implant (Pilatus Swiss Dental GmbH, Egolzwil, Switzerland) (21, 77%) 5 different implant brands were used. No statistically significant relationship was observed in the evaluation of the brands used according to the procedures applied (chi-square test) (p = 0.389).

### The effect of implant characteristics (diameter / length), smoking and age on immediate implantation success

Of the 124 implants placed in 69 patients included in the study, 116 (95.55%) were successful, while 8 (4.45%) were unsuccessful. The mean diameter of the unsuccessful implants was lower than the successful ones, but no statistically significant relationship was observed (p = 0.0953). No statistically significant relationship was observed between the success of the implants and their height (p = 0.276). While the mean number of daily cigarette smoking was 16.88 ± 12,229 in unsuccessful immediate implants, this number was 7.07 ± 9.647 in successful patients. A statistically significant relationship was observed between success and the number of daily cigarettes (p = 0.007). No significant relationship was found between age and success (p = 0.345) (Table 2).

**Table 2:** The Relationship Between Implant Success, Implant Characteristics, Smoking and Age

Variables		Number of Implants	Mean	Std. Deviation	P Value
Diameter	Fail	8	4,188	0,4357	0,953
	Success	116	4,177	0,5024	
Length	Fail	8	9,875	2,0310	0,276
	Success	116	10,638	1,8992	
Smoking (Number/Day)	Fail	8	16,88	12,229	0,007 *
	Success	116	7,07	9,647	
Age	Fail	8	43,50	7,783	0,345
	Success	116	47,16	10,695	

### The relationship between implant characteristics (diameter / height), smoking, age and gender

Of the 124 implants placed in 69 patients included in the study, 47 (37.90%) were applied to women, while 77 (62.1%) were applied to men. The mean diameter of the implants applied to men is observed to be smaller than those applied to women (4.143 ± 0.485). The mean height of implants applied to men (10.74 ± 1.895) was longer than those applied to women (10.340 ± 1.926). No significant correlation was found between the diameter and length of the implants and gender (p = 0.323), (p = 259). No significant relationship was observed between gender and age (p = 0.562), a significant relationship was observed between gender and daily smoking. While the number of daily cigarettes used in women is 2.128 ± 5.50, this number is 11.104 ± 10,688 in men (p = 0.000) (Table 3).

**Table 3:** Relationship between Implant Characteristics (Diameter / Height), Smoking, Age and Gender

Variables		Number of Implants	Mean	Std. Deviation	P Value
Diameter	Female	47	4,234	0,516	0,323
	Male	77	4,143	0,485	
Length	Female	47	10,340	1,926	0,259
	Male	77	10,740	1,895	
Smoking (Number/Day)	Female	47	2,128	5,590	0,000 *
	Male	77	11,104	10,688	
Age	Female	47	46,213	10,428	0,562
	Male	77	47,351	10,661	

### Distribution of smoking, gender and implant success by groups

Cross-tables and chi-square test were used to evaluate the relationship of smoking and gender with implant success between groups. Of the 8 failed implants, 2 were in Group 1, 2 were in Group 2, and 4 were in Group 3. Of the 71 implants in Group 1, 2 were unsuccessful and 69 were successful. The success rate is 97.2%. Of the 31 implants in Group 2, 2 were unsuccessful and 29 were successful. The success rate is 93.5%. Of the 22 implants in group 3, 4 were unsuccessful and 18 were successful. The success rate is 81.8%. A significant correlation was found between the study groups and implant success in terms of χ2 test (p = 0.037). Of the 8 failed implants, 1 consisted of non-smokers and 7 of them were smokers. While the success rate in the non-smoking population is 98.5%, the success rate in the smoking population is 87.7%. A significant correlation was found between smoking and implant success in terms of χ2 test (p = 0.015). No statistically significant relationship was found between the gender factor and implant success (p = 0.126) (Table 4).

**Table 4:** Distribution of Smoking, Gender and Implant Success by Groups

Variables		Group 1	Group 2	Group 3	Total	P Value
Implant Success	Fail	2 (%2,8)	2 (%6,5)	4 (%18,2)	8 (%6,5)	0,037 *
	Success	69 (%97,2)	29 (%93,5)	18 (%81,8)	116 (%93,5)	
	Total	71 (%100)	31 (%100)	22 (%100)	124 (%100)	
	Variables		No smoking	Smoking	Total	P Value
Implant Success	Fail		1 (%1,5)	7 (%12,3)	8 (%6,5)	0,015 *
	Success		66 (%98,5)	50 (%87,7)	116 (%93,5)	
	Total		67 (%100)	57 (%100)	124 (%100)	
	Variables		Female	Male	Total	P Value
Implant Success	Fail		1 (%2,1)	7 (%9,1)	8 (%6,5)	0,126
	Success		46 (%97,9)	70 (%90,9)	116 (%93,5)	
	Total		47 (%100)	77 (%100)	124 (%100)	

## Discussion

According to the current research results, Satisfactory survival rates (97.2%) were observed in Group1 patients who did not undergo an additional procedure such as GBR or lateral approach MSL, similar to the literature [8, 11, 28]. Although the survival rate of Group 2 was lower than that of Group 1, the success rate in Group 2 patients was at acceptable levels (93.5%). In Group 3 patients who underwent lateral approach MSL procedure in addition to immediate implant applications to extraction sockets with periapical pathology, the success rate was observed to be relatively low (81.8%). The mean length of the implants used in Group 1 and Group 2 patients was statistically significantly longer than Group 3. In addition, the average diameter of the implants used in Group 2 and Group 3 patients is observed to be thicker than Group 1. When the results were evaluated, no effect was observed on the success rates of the factors such as gender, age, diameter and height of the implants used. No significant relationship was observed between the diameter and length characteristics of the implants used and gender.

When the current studies in the literature are evaluated, it is proven that immediate implant applications to infected or uninfected extraction sockets are a very successful approach type [11, 12]. Compared to the conventional method, immediate implant applications offer advantages such as reducing the number of surgical procedures, clearly defining the boundaries of the extraction socket, placing the implant in the most appropriate position, stabilizing the bone and soft tissue [29]. Besides the existing advantages, a problem encountered is the gap between the coronal part of the implant and the extraction socket. The use of bone grafts and barrier membranes is recommended for preservation of hard and soft tissue architecture and stabilization of socket walls [30]. When the literature data is evaluated, there is no clear consensus on the grafting of the spaces between the implant and the socket walls and the type of graft that should be used in immediate implant applications [29]. In our current study, a mineralized cancellous bone allograft was used to fill the spaces larger than 2 mm between the implant neck and the socket wall in all patients who underwent GBR, and all allografts were covered with a collagen membrane. The success rate in the GBR applied group (Group 2) was 93.5%. Although lower success rates were observed when compared to Group 1, it is clear that there is a satisfactory success rate when compared with the articles in the literature [8, 12, 28].

Posterior maxilla is seen as a risky area in clinical implantology practice with atrophic alveolar crests and limited residual bone height. MSL procedures are routinely performed with or without the use of bone grafts to achieve the minimum bone height required for placement of dental implants [31]. MSL procedures can be performed as a single or two-stage procedure that includes the lateral window approach, crestal approaches and their modifications [25, 32].

In cases where the distance between the floor of the maxillary sinus and the alveolar crest is 8 mm or more, a standard length implant can be placed without the need for MSL procedures. For residual bone heights between 5 and 8 mm, the use of short implants is among the minimally invasive treatment methods that can be preferred. In this case, another option is sinus lifting applications with crestal approach. In cases where the residual bone height is less than 5 mm, the general trend is lateral approach MSL procedures [25].

In our clinical practice, short implant use or sinus lifting procedure with crestal approach is preferred in cases where the residual crestal bone amount is between 5 and 8 mm after tooth extraction with periapical pathology. A 2–3 mm height gain with the crestal approach provides sufficient space for the placement of an 8 mm normal-length implant. The use of additional bone grafts was not required in patients in whom the crestal approach was performed and these patients were not included in Group 3.

In cases where the crestal bone height was less than 5 mm, the lateral approach MSL procedure was applied. When similar studies in the literature are examined, the absence of another study in which tooth extraction with periapical pathology was performed, socket debridement was provided, the lateral approach MSL procedure was performed and the dental implant was placed simultaneously constitutes an obstacle to comparing the survival rates in Group 3 with the literature data. The complexity of the procedure, the risk of error at every stage, and the high smoking rates observed in the included patients may have resulted in a lower success rate (81.8%) compared to immediate implant studies. In addition, the small number of patients compared to other groups (22 patients) is among the main limitations of the study.

Tobacco and smoking reduce leukocyte activity, which is responsible for low chemotactic migration rate, low motility levels, and low phagocytic activity. This causes a decrease in infection resistance and delays in wound healing [33]. In addition, smoking has been associated with lower calcium absorption. One possible mechanism by which cigarette smoking may affect osteointegration is decreased blood flow as a result of increased peripheral resistance and inhibition of platelet aggregation. Tobacco use directly affects osteoblast function and its toxic by-products delay wound healing. It also inhibits cell proliferation [34]. Literature data show that the survival rate of dental implants is lower in smokers [35]. In a systematic review-meta-analysis published by Strietzel et al. in 2007, it was concluded that the risk of biological complications is significantly increased among smokers, an important risk factor for dental implant treatment and accompanying augmentation procedures [36]. Our present study supports these findings. There is a significant difference in survival rates between the smoking population and the non-smoking population. While the survival rate is 87.7% in the smoking population (7 failed implants), a 98.5% success rate (1 failed implant) is observed in the non-smoker population. Of the total 8 failed implants, 7 were among the smokers, 4 of these 7 failed implants were in Group 3 patients, 2 were in Group 2 patients, and 1 were in Group 1 patients. Especially in groups where the surgical procedure is complex, smoking seriously affects survival rates. As an additional data, male patients were observed statistically significantly higher among the smoking population.

Among the limitations of the study; the inhomogeneity of patient distribution between groups, the use of different implant brands, the 18-month follow-up period after the completion of prosthetic restorations, the inability to identify bacterial features of the implant socket in failed implants, the inhomogeneity of smoking among groups, and the use of a single graft and membrane type in GBR-lateral approach MSL procedures can be shown. In particular, the implant survival rates observed in Group 2 and Group 3 need to be supported by prospective studies with larger sample sizes. In addition, there is a need for more comprehensive studies comparing the success rates of the groups in the current study with implants placed in areas where the physiological healing process is completed after tooth extraction and implants placed in fresh extraction sockets without periapical infection.

## Conclusions

When the results of the study are evaluated;

- High implant survival rates are observed if adequate curettage and debridement are provided to the sockets with periapical pathology.
- Implant survival rates observed in GBR applications combined with immediate implant placement in extraction sockets with periapical pathology are at acceptable and satisfactory levels.
- The survival rates observed for immediate implant placement in extraction sockets with periapical pathology simultaneously with lateral approach MSL procedures are relatively low. However, this result should be evaluated considering the limitations of the study.
- Smoking is an important risk factor for immediate implant surgery and augmentation procedures.
- Gender factor, age, characteristics such as diameter and height of the implants used do not have an effect on the success rates. There is no significant relationship between the diameter and length characteristics of the implants used and gender.

## Data Availability

This research was conducted with the consent of all participants. The data set supporting the findings of the study can be obtained from the corresponding author upon appropriate requests.
